# A post-traumatic ossified subdural chronic hematoma successfully managed in a 34-year-old woman: a case report

**DOI:** 10.1097/MS9.0000000000000304

**Published:** 2023-03-25

**Authors:** Marah Mansour, Mohammad Adi, Hana Mousa, Nada Haroun, Muhamad Rustum, Mohamad Joha, Mohammed Abdulrahman

**Affiliations:** a Faculty of Medicine, Tartous University, Tartous; b Faculty of Medicine, Damascus University; c Faculty of Medicine, Syrian Private University, Damascus; d Department of Neurosurgery, Tishreen University Hospital, Lattakia, Syrian Arab Republic

**Keywords:** calcified, case report, chronic subdural hematoma, ossified, trauma

## Abstract

Ossified or calcified subdural chronic hematoma is an uncommon condition with an incidence of 0.3–2% of all chronic subdural hematomas. It can cause significant mortality and morbidity, especially in young patients. Because of the rare incidence of this condition, its pathophysiology and management preferences have not yet been clearly established, which raises the importance of reporting such cases in order to add valuable databases to the literature.

**Case Presentation:**

A 34-year-old woman with a history of head trauma several years ago presented with refractory headaches, convulsions, and muscle weakness. Computed tomography showed an extra-axial calcified lesion in the frontal lobe. Surgical treatment was decided considering the patient’s age, in addition to the presence of serious medically uncontrolled symptoms. The calcified lesion was successfully removed surgically, and the patient recovered completely. Pathological examination confirmed the diagnosis of ossified subdural chronic hematoma.

**Clinical Discussion:**

The symptoms of ossified subdural hematomas are not specific. However, the presence of a history of head trauma should raise suspicion of this condition. Computerized tomography is usually used as the diagnostic method. Nevertheless, it is unable to differentiate ossified chronic subdural hematomas from other intracranial extra-axial calcified lesions that need to be considered as a differential diagnosis. Pathologic investigations are needed to provide the final diagnosis.

**Conclusions:**

We highly recommend surgical therapy for ossified subdural hematomas that are symptomatic and persistent, especially in young patients. We further stress the significance of postsurgical anticonvulsant prophylaxis, particularly in patients presenting convulsions.

## Background

Highlights

- Ossified subdural chronic hematoma is a rare condition that can arise years after trauma.
- The symptoms are similar to those of any other brain space-occupying lesions.
- Imaging diagnostic studies reveal the presence of calcified lesions.
- Diagnosis can be confirmed only with pathology.
- Surgical treatment should be done in selected cases.

Chronic subdural hematoma (CSDH) is a prominent clinical issue in neurosurgery that is commonly thought of as a traumatic complication^1^. Ossified subdural chronic hematoma (OSCH) is a very rare manifestation that occurs in only 0.3–2% of CSDHs. Children and young adults are more likely to have OSCH than older people, and OSCH is usually observed in post-traumatic subdural hematomas (SDHs)^2^,^3^. The pathophysiology of OSCH is not fully understood; poor circulation and absorption lead to stasis of the subdural fluid due to thick surrounding membranes, venous thrombosis, the prolonged presence of hematoma, the metabolic predisposition for calcification, and poor venous outflow are factors that may be implicated in the pathogenesis of OSCH. Ossification is the final step in the development of CSDHs, preceded by hyalinization and calcification. As a result, calcification takes a few months to occur, whereas ossification takes many years, and the term ‘OSCH’ should only be used for hematomas that show signs of bone development^3^. There are variances in the symptoms and findings. The research has described asymptomatic cases as well as those with increased intracranial pressure and severe symptoms. In addition to headache being the most common symptom, lethargy, disorientation, amnesia, hemiparesis, gait abnormalities, seizures, and mental retardation may be noticed^4^. MRI and computed tomography (CT) are necessary to make the diagnosis. However, surgical findings and pathological examinations are needed to confirm the diagnosis. Surgical treatment is required in symptomatic and young patients^4^,^5^. We report a case of OSCH in a 34-year-old woman after old head trauma caused by a car accident.

## Case presentation

**Figure 1:**
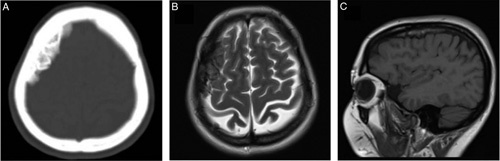
(A–C) A computed tomography scan showing an extra-axial calcified lesion in the right frontal lobe near the calvaria (A). MRI studies in T2A (B) and T1A (C) show hypointense lesions compatible with calcification/ossification.

A 34-year-old woman was admitted to the Department of Neurosurgery with a complaint of persistent headaches and convulsions. The patient had no medical or family history. The patient was injured in a car accident 7 years before the admission date, which resulted in a nondisplaced right parietal linear fracture and a mandibular fracture without intracranial bleeding or neurological sequelae, and was treated conservatively. After the trauma, the patient had chronic headaches relieved by painkillers. However, a year ago, headaches became refractory, and the patient started complaining of recurring convulsions, which were followed by transient left hemiplegia (Todd’s paralysis). Convulsions’ frequency gradually increased despite pharmacological treatment that reached maximum doses without improvement. Muscle weakness had become permanent with muscle strength of 4/5 on the left side during the last month before the surgery. Brain CT revealed an extra-axial calcified lesion in the right parietal frontal lobe near the calvaria, and the MRI in T2A and T1A (Fig. 1A–C) showed a hypointense lesion compatible with calcification/ossification.

**Figure 2:**
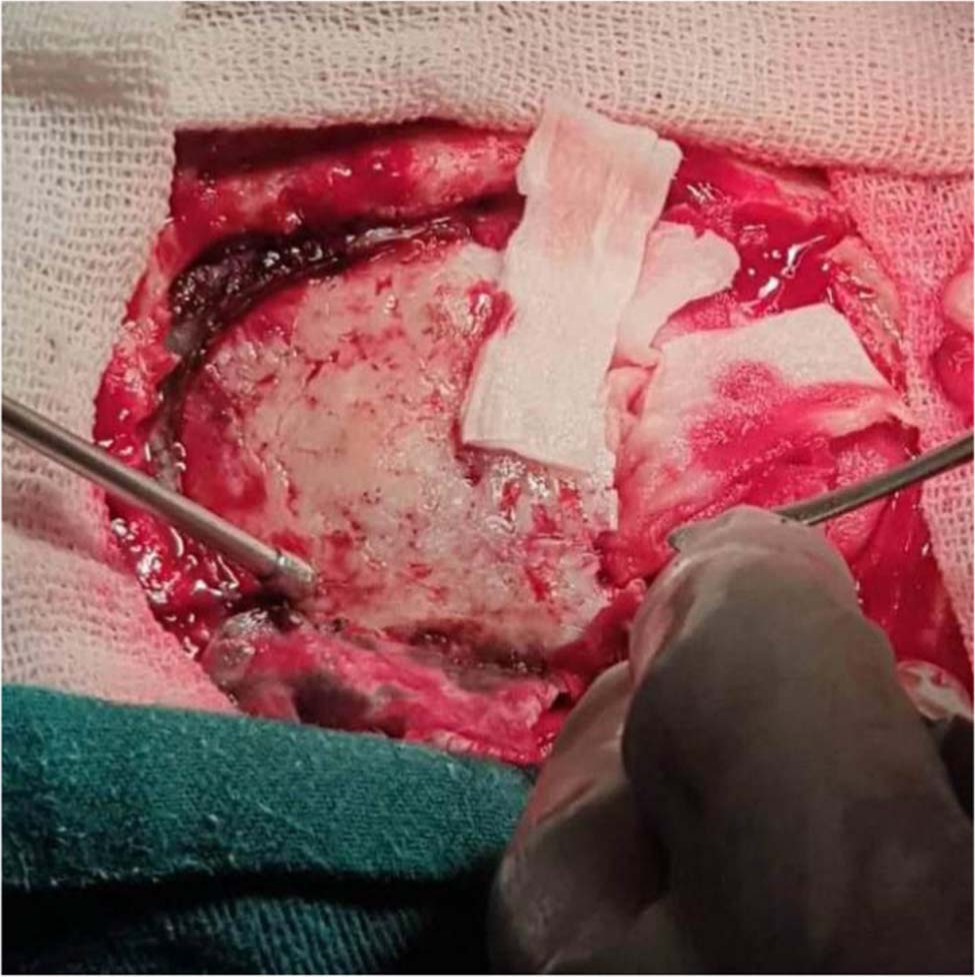
During surgery, after bone lifting and dura dissection, the lesion is shown as a whitish, hard surface. The calcified hematoma was removed after a bone portion was raised and the dura mater was split open.

The diagnosis of post-traumatic ossified or calcified CSDH was suspected based on the patient’s history of head trauma, physical examination, and imaging findings. Surgery was decided upon considering the patient’s young age, the presence of refractory convulsions and muscle weakness, as well as the patient’s desire to undergo surgery that would relieve symptoms permanently. The surgery was carried out in a general university hospital’s neurosurgery unit. Two junior trainees with 3 years of surgical specialty training worked under the leadership of a neurosurgery specialist with 25 years of experience. The procedure was done while the patient was under general anesthesia. During surgery, after bone lifting, dura dissection and lesion isolation were done easily since the lesion was of a hard consistency without adhesions to the surrounding tissues (Fig. 2).

**Figure 3:**
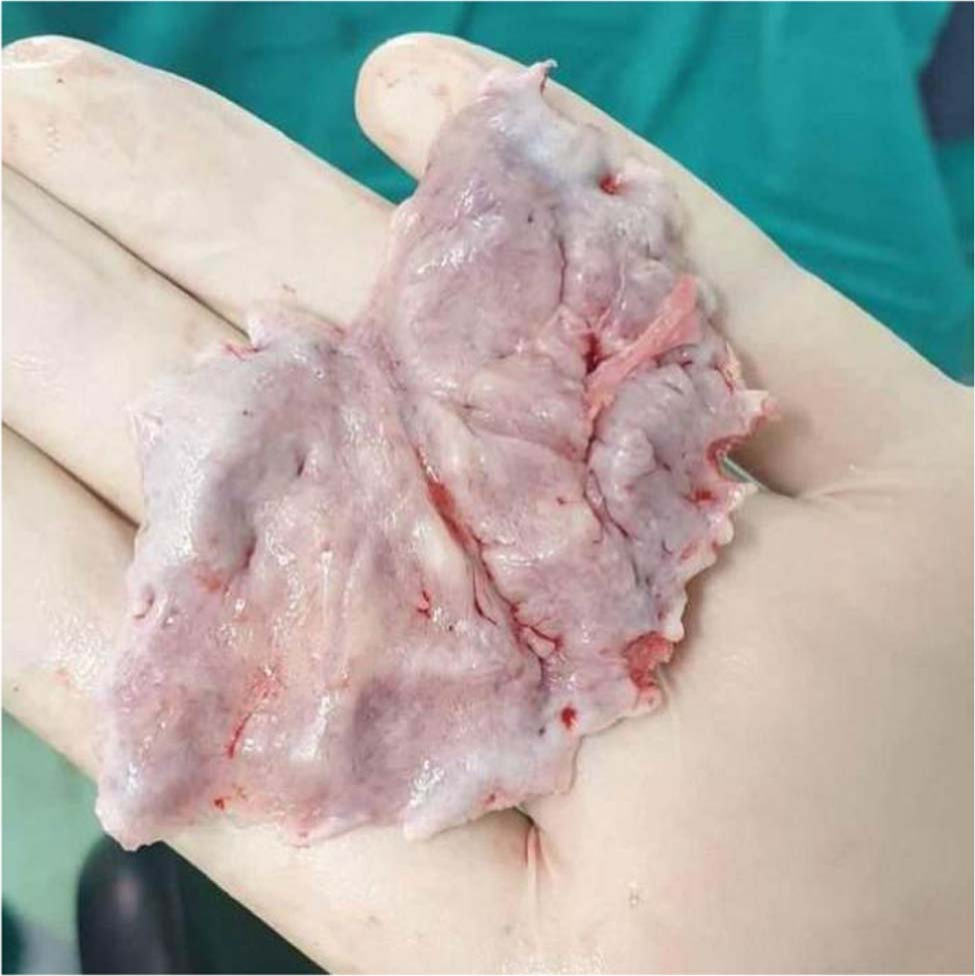
The extracted lesion was hard and measured 6.3×1.1×7.3 cm.

Then the lesion was carefully dissected without injuring the brain. The extracted lesion was hard and measured 6.3×1.1×7.3 cm (Fig. 3).

**Figure 4:**
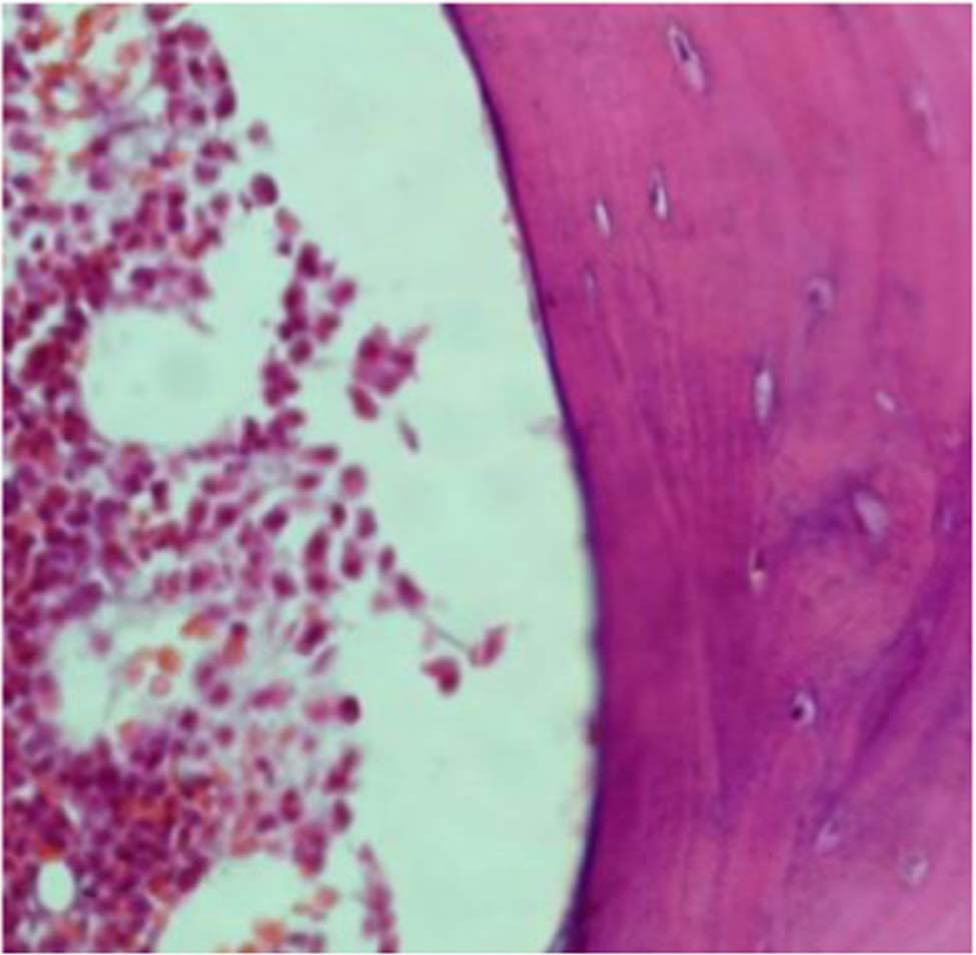
The pathological smear showing trabeculae of lamellated bone with marrow.

Following the surgery, no complications occurred during the recovery period, and the patient was discharged with an improvement in muscle strength, which was 5/5 at discharge. A CT scan performed 5 days after the surgery revealed no abnormalities. Histopathologically, the lesion was reported as bone tissue with trabeculae of lamellated bone and bone marrow (Fig. 4) so, the diagnosis of OSCH was confirmed.

It was decided that the patient should continue using the anticonvulsant treatment for at least 1 year, with follow-up visits to ensure improvement and tapering the anticonvulsant dose when appropriate. In the first 6 months after surgery, no convulsions occurred, then the patient abruptly stopped taking the anticonvulsant medication by herself. Two weeks later, she experienced a seizure. The anticonvulsant treatment was reestablished and planned to be continued for a further 2 years with a gradually tapering dose.

This case report has been reported in line with the SCARE (Surgical CAse REport) Criteria^6^.

## Discussion and conclusions

SDHs are defined as chronic when they occur more than 3 weeks after an initial insult, which is commonly a traumatic head injury^5^,^7^. After at least 6 months, calcification may frequently occur, leading to calcified CSDHs. After many years, calcification may rarely transform into ossification^1^,^5^. Therefore, calcification and ossification should not be considered the same entity since ossified hematomas must involve the formation of bone tissue, not just calcification. When calcification or ossification occurs bilaterally or covers a significant portion of the brain surface, it is called an ‘armored brain’ or ‘Matrioska head.’ In 1884, von Rokitansky was the first to publish OSCH autopsy findings. While Goldhan performed the first surgical excision of OSCH, which was documented in 1930^3^,^4^. In contrast with other types of SDHs, which are common in the practice of neurosurgery and more prevalent among elderly patients, OSCH typically occurs in children and young adults, as in this case, and is a much rarer entity^1^,^2^. It is reported that only 0.8–10% of CSDH patients develop OSCH^8^. As a consequence, there are no shared or consistent beliefs regarding the clinical approach or the pathophysiology^4^. While OSCH is typically seen after trauma, other nontraumatic conditions may present with it, as in postmeningitic patients and long after ventriculoperitoneal shunts^3^,^4^. In our case, there was a documented history of head trauma and no other insults to the brain. Based on some research, the pathogenesis of calcified CSDH may be influenced by vascular malformations, coagulopathy, alcoholism, intracranial hypotension, and primary or metastatic malignancies^1^. In this patient, there were none of these risk factors or any past medical or surgical history. The pathogenesis of calcification and ossification is not yet understood. Furthermore, it has been proposed that the local poor metabolic state, circulation, and absorption in the subdural space play a role in the calcification process^2^. There are reported cases of bilateral CSDHs, but only unilateral calcification. This supports the hypothesis that local factors may contribute to the process of calcification^4^,^5^. Clinically, OSCH patients are usually asymptomatic. However, when OSCH is symptomatic, complaints are not unique and are similar to those of nonossified or noncalcified SDH or any other space-occupying lesion^2^,^8^. Headache is reported as the most common symptom, followed by confusion, drowsiness, dementia, and convulsions. Brain atrophy is assumed to be the reason behind asymptomatic cases in elderly patients. On the other hand, headaches are frequently experienced by young individuals because they are more susceptible to increasing intracranial pressure^1^,^2^. As in the case of our patient, a young person who has endured years of minor chronic headaches but has waited to seek medical attention until convulsions occurred. The preferred method for determining the location and age of most SDHs is CT. But, in cases of bilateral CSDH, MRI is superior to CT^7^. However, pathological investigations and intraoperative assessment are necessary to confirm the diagnosis^5^. By demonstrating the presence of bone formation, pathology represents the definitive diagnostic method for distinguishing calcified from ossified hematomas, as well as other intracranial calcified lesions^3^. In this case, the CT was sufficient to guide us toward the diagnosis, and the MRI was used to exclude other entities that may have similar presentations (Fig. 1A–C). Although the definitive diagnosis was given by the pathology that confirmed the presence of bone tissue in the lesion (Fig. 4). Histological diagnosis is needed because calcified extra-axial space-occupying pathologies such as calcified subdural empyema, epidural hematoma, calcified arachnoid cyst, malignant tumors, and meningiomas, as well as cranial bone tumors, may mimic the radiological presentation of calcified and OSCH and should be considered in the differential diagnosis^7^,^9^,^10^. Due to the possibility of cortical damage, ossified SDH is typically treated conservatively in elderly or asymptomatic patients. However, it is believed that surgical therapy is required for young or symptomatic patients^8^. So the surgery decision for this patient was not arguable because of the presence of symptoms that were not controlled by medical treatment, and there was no other explanation for them, as well as considering the young age of the patient. When the OSCH is removed surgically, it reduces the mass effect and enhances cerebral blood flow, resulting in neurological improvement. Extreme care should be taken during surgical dissection because the lesion may be connected to the adjacent brain tissue. In the event of dense adhesions to the brain parenchyma, partial resection may be the only choice. Partial resections restrict brain re-expansion, which increases the patient’s chance of developing a hematoma^3^,^5^,^8^. A clear plane between the ossified hematoma and the brain was noticed in our patient, which was used to achieve complete resection without damaging the brain (Fig. 3). Rather than brain injury, surgical treatment may end with many complications. One of the most frequently reported complications is recurrent hemorrhage, which should be differentiated from the residual fluid that may need months after surgery to be resolved^4^. One rare but fatal complication is the development of tension pneumocephalus, which needs urgent intervention in contrast with recurring hemorrhage or residual fluid that needs follow-up by control CT, with intervention only needed in clinically deteriorating cases^4^,^8^. Our patient’s follow-up CT was clear, clinical improvement was proven radiologically, and further follow-up was planned. About 10% of patients with OSCH report having seizures after surgery, so patients who undergo surgery for this condition should be given an antiepileptic medication as a preventative measure, although there is an ongoing debate in the literature over the effectiveness of anticonvulsant therapy^7^. The episode of convulsions that our patient suffered after stopping the anticonvulsant may be explained by the abrupt stopping of the medication, but it also implies the importance of prophylactic anticonvulsant treatment after surgery. In conclusion, a long time after the development of a SDH, a hematoma membrane may become calcified or even ossified. However, calcification and ossification are not the same and should be differentiated depending on the histological features, as well as addressing the two conditions carefully in the literature without using the two terms synonymously. This report indicated that surgical therapy is strongly advised for patients who have symptoms of OSCH and that young people may be more susceptible to developing the condition. It also suggests the significance of prophylactic anticonvulsant therapy following surgery.

## Ethical approval

Not applicable.

## Patient consent

Written informed consent was obtained from the patient for publishing this case report and any accompanying images. A copy of the written consent is available for review by the Editor-in-Chief of this journal on request.

## Sources of funding

No funding sources.

## Author contribution

M.M.: design of the study, data collection, data interpretation and analysis, drafting, and critical revision; M.A.: data collection, data interpretation and analysis, critical revision, and drafting; H.M. and N.H.: data interpretation and analysis, critical revision, and drafting; M.R. and M.J.: critical revision and drafting; M.A.: the supervisor, patient care, drafting, and critical revision. All authors were involved in the approval of the final manuscript.

## Conflicts of interest disclosure

The authors declare that there are no conflicts of interest.

## Guarantor

Dr Mohammed Abdulrahman is the guarantor of this work.

## Provenance and peer review

Not commissioned, externally peer-reviewed.
